# The Fibroblast Growth Factor Receptor 2 p.Ala172Phe Mutation in Pfeiffer Syndrome—History Repeating Itself

**DOI:** 10.1002/ajmg.a.35842

**Published:** 2013-03-26

**Authors:** Sally Jay, Akira Wiberg, Marc Swan, Tracy Lester, Louise J Williams, Indira B Taylor, David Johnson, Andrew OM Wilkie

**Affiliations:** 1 Department of Plastic Surgery, John Radcliffe HospitalOxford, UK; 2 Molecular Genetics Laboratory, Churchill HospitalOxford, UK; 3 Weatherall Institute of Molecular Medicine, University of Oxford and Oxford Craniofacial Unit, John Radcliffe HospitalOxford, UK; 4 Department of Plastic Surgery and Oxford Craniofacial Unit, John Radcliffe HospitalOxford, UK

**Keywords:** craniosynostosis, Pfeiffer syndrome, FGFR2 A172F mutation, selfish spermatogonia

## Abstract

Pfeiffer syndrome is an autosomal dominant condition classically combining craniosynostosis with digital anomalies of the hands and feet. The majority of cases are caused by heterozygous mutations in the third immunoglobulin-like domain (IgIII) of FGFR2, whilst a small number of cases can be attributed to mutations outside this region of the protein. A mild form of Pfeiffer syndrome can rarely be caused by a specific mutation in FGFR1. We report on the clinical and genetic findings in a three generation British family with Pfeiffer syndrome caused by a heterozygous missense mutation, p.Ala172Phe, located in the IgII domain of FGFR2. This is the first reported case of this particular mutation since Pfeiffer's index case, originally described in a German family in 1964, on which basis the syndrome was eponymously named. Genetic analysis demonstrated the two families to be unrelated. Similarities in phenotypes between the two families are discussed. Independent genetic origins, but phenotypic similarities in the two families add to the evidence supporting the theory of selfish spermatogonial selective advantage for this rare gain-of-function FGFR2 mutation. © 2013 Wiley Periodicals, Inc.

## INTRODUCTION

Pfeiffer syndrome classically describes a combination of craniofacial and limb anomalies. Multisuture craniosynostosis, exorbitism, and midface hypoplasia are common craniofacial features. Radially deviated broad thumbs and broad great toes are typical extracranial features and less frequently, partial syndactyly in the hands and feet may be present [Anantheswar and Venkataramana, 2009]. There is however significant variation in phenotype and cases have been described of Pfeiffer syndrome without craniosynostosis [Hackett and Rowe, 2006]. The phenotypic diversity relates, in part, to the genetic heterogeneity.

Pfeiffer syndrome is an autosomal dominant condition with an incidence of approximately 1 in 120,000 births. It is caused by heterozygous mutations in the fibroblast growth factor receptors types 1 and 2 (FGFR1 and FGFR2) [Johnson and Wilkie, 2011]. Occasionally, a specific mutation in FGFR1, p.Pro252Arg can cause Pfeiffer syndrome—phenotypically these families have classic hand and foot anomalies with variable presence of craniosynostosis and generally milder craniofacial features [Muenke et al., 1994; Rossi et al., 2003; Hackett and Rowe, 2006]. More frequently, FGFR2 is implicated and more than 40 different heterozygous mutations causing Pfeiffer syndrome have been identified in *FGFR2* [Wilkie, 2008]. Ninety-four percent of these mutations occur in either exon 8 or exon 10, encoding the third immunoglobulin-like domain of the protein (IgIII), but mutations in seven different exons outside this hotspot have also been identified [Kan et al., 2002; Lajeunie et al., 2006].

*FGFR2* encodes a protein involved in cell division and regulation of cell growth and maturation, affecting processes such as embryonic development, formation of blood vessels, and wound healing. Specifically, this protein is a transmembrane receptor tyrosine kinase comprising an extracellular ligand-binding region (IgI, IgII, and IgIII), a single pass transmembrane region and a split tyrosine kinase domain. Mutations in *FGFR2* lead to predominantly missense substitutions in the amino acid sequence resulting in a gain-of-function. Of the mutations that have occurred outside the main hotspot region, only a single instance has been identified in exon 5, which encodes part of the IgII domain. This mutation involved substitution of two consecutive nucleotides (c.514_515delGCinsTT, encoding p.Ala172Phe) and was previously known only from Pfeiffer's index case, a three-generation German family that he described in 1964 and was associated with an atypical phenotype [Pfeiffer, 1964; Kan et al., 2002]. Here we report an additional, independent, three-generation British family found to have the identical mutation, and compare the phenotypes and genetic backgrounds of the two families.

## CLINICAL REPORT

### Phenotypic Analysis of the British Family

A 6-month-old boy (proband) was referred to the Oxford Craniofacial Unit at the request of his mother and maternal grandfather, both of whom had previously been told they had Pfeiffer syndrome. He had been born at 38 weeks by forceps assisted delivery, following an uncomplicated pregnancy. Antenatal ultrasound scans had raised the concern of abnormal head shape; however this was not evident at birth and the anomalies were predominantly confined to the hands and feet.

**FIG. 1:**
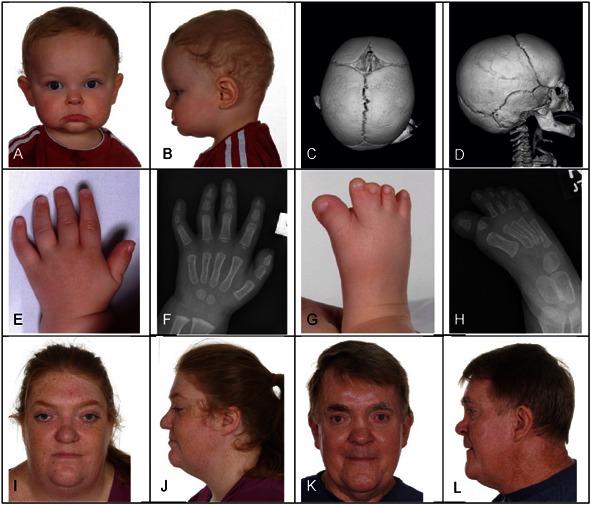
Images of British family showing craniofacial and limb features with similarity in phenotype and severity. **A**–**H**, proband: (A,B)—AP and left lateral photographs (note mild dysmorphic features and hypertelorism) (C,D)—vertex and right lateral 3D CT head (note presence of all sutures) (E,F)—photograph and X-ray of left hand (note broad radially deviated thumb, 3/4 mild incomplete syndactyly and little finger clinodactyly) (G,H)—photograph and X-ray of right foot (note broad medially deviated great toe, 2/3 complete, 4/5 incomplete syndactyly and absence of middle phalanges). **I**,**J**: Proband's mother: AP and left lateral photographs (note dysmorphic features and hypertelorsim). **K**,**L**: Proband's maternal grandfather: AP and left lateral photographs (note mildly dysmorphic features, hypertelorism, and flattened nasal bridge). [Color figure can be seen in the online version of this article, available at http://wileyonlinelibrary.com/journal/ajmga]

On examination, the proband (Fig. 1A–H) was developmentally normal. He had mildly dysmorphic features, hypertelorism, and a high arched palate. However, he had no midface hypoplasia and a normal looking head shape; although, when measured he was mildly brachycephalic with a cephalic index (CI) of 85%. He had a normal anterior fontanelle, no sutural ridging, and no clinical evidence of craniosynostosis. A computerized tomography (CT) scan confirmed no evidence of craniosynostosis. Examination of the feet showed broad, medially deviated great toes with 2/3 complete and 4/5 incomplete simple syndactyly bilaterally. Radiological examination also showed absence of the middle phalanges of the toes. The hands showed bilateral broad, radially deviated thumbs, 3/4 mild incomplete simple syndactyly, and little finger clinodactyly.

The proband's mother (Fig. 1I,J) also showed no clinical evidence of craniosynostosis and her CI was 79%. She did however have dysmorphic features, hypertelorism, midface hypoplasia, a high arched palate, and reported previous mandibular surgery for malocclusion. Examination of the right foot showed broad great and little toes with 2/3 complete and 3/4/5 incomplete presumed simple syndactyly. The left foot had broad great, 4th and little toes and 2/3/4 incomplete presumed simple syndactyly. The hands had bilateral broad, radially deviated thumbs with no interphalangeal joint flexion and 2/3/4 incomplete presumed simple syndactyly. There was no clinodactyly.

The proband's maternal grandfather (Fig. 1K,L) again showed no evidence of craniosynostosis and his CI was also 79%. He did have mildly dysmorphic features, hypertelorism, midface hypoplasia, and a high arched palate. Examination of the feet showed all toes were broad with 2/3/4/5 presumed simple syndactyly bilaterally. The hands had bilateral broad thumbs and scarring consistent with previous 2/3/4 syndactyly release.

### Genotypic Analysis of the British Family and Comparison With the German Family

In the British family, DNA samples were obtained from the affected child, his affected mother and his affected maternal grandfather. DNA samples from Pfeiffer's original German family were already available to us [Kan et al., 2002].

**FIG. 2:**
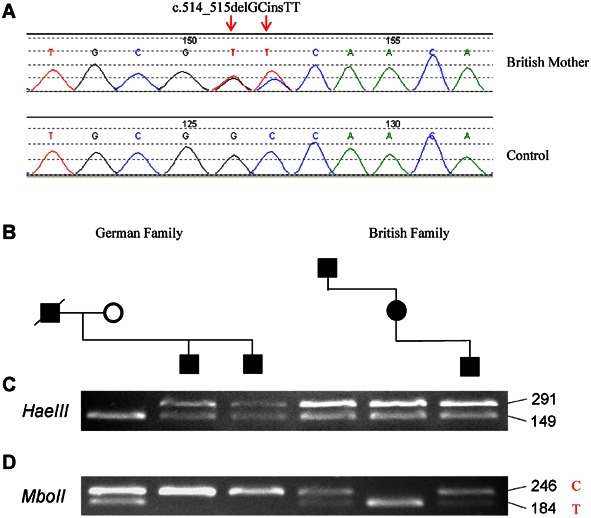
Genotypic analysis of the British and German Families. **A**: DNA sequencing of British proband's mother showing heterozygosity of two adjacent nucleotides (shown by arrows) compared with control DNA. **B**: Simplified pedigrees of the British and German families, showing the biological relationships of the samples analyzed. **C**: *HaeIII* restriction enzyme digest showing heterozygosity for the normal (149 bp) allele in affected individuals, which can only occur when the c.514G > T and c.515C > T are present in *cis*. **D**: Analysis of C/T SNP rs2981432 by *MboII* digestion showing that in the German family, the mutation segregates with the C allele of the SNP (C), whereas in the British family the mutation segregates with the T allele (T). [Color figure can be seen in the online version of this article, available at http://wileyonlinelibrary.com/journal/ajmga]

DNA sequencing of exon 5 of *FGFR2* in the mother of the British family was performed, demonstrating heterozygosity at two adjacent nucleotides shown in Figure 2A. Restriction digestion with *HaeIII* demonstrated the same mutation in all three affected individuals, and revealed that the normal sequence (GGCC) was preserved on one allele (Fig. 2C), showing that the two mutations were present in *cis*: c.514_515delGCinsTT, encoding p.Ala172Phe. This sequence change is identical to that previously described in Pfeiffer's original family [Kan et al., 2002].

To establish whether the unusual double mutation present in the two families had a single mutational origin (because of an unknown distant genealogical relationship) or independent origins, we typed selected individuals from both families (see Fig. 2B for pedigrees) for known polymorphic sequence changes flanking the site of the mutation. Initial genotyping of an informative microsatellite locus [Goriely et al., 2010] 27.3 kb in a 3′ direction (*D10S1483*), revealed that in the German family the disease-causing mutation segregated with a 140 bp allele; in contrast, in the British family the disease-causing mutation segregated with a 142 bp allele, suggesting that the p.Ala172Phe mutation had arisen independently in the two families (data not shown). However, we could not exclude the small possibility of either a length mutation of the microsatellite sequence or recombination between the microsatellite and exon 5 occurring in an ancestral generation. Therefore, to corroborate this result, we used phased haplotype data (PhaseIII, release #2, Feb. 09) obtained from HapMap [The International HapMap 3 Consortium, 2010] to identify potentially informative single nucleotide polymorphism (SNP) variations close to the site of the mutation. Amongst 10 SNPs genotyped, in 5 the pedigree structure in one or both families did not enable the disease-associated allele of the SNP to be assigned unambiguously. For a further four SNPs (rs1047057, rs1649200, rs3135772, and rs2981451), the mutation was present on the same allele in both families, representing an identical G-A-C-T haplotype (data not shown). However, genotyping was also informative using the final SNP rs2981432, which is located 15.7 kb 5′ of the mutation, and was analyzed by PCR amplification using primers 5′-CTAGTTGGCATCTGGGGCTTGGCATGC-3′ and 5′-ACCAAATCAGGGCAGGATCAAAGGCAACTG-3′ followed by *MboII* digestion. This showed that in the German family, the C allele is present in *cis* with the mutant *FGFR2*, whereas in the British family, it is the T allele (Fig. 2D). As discussed below, this result, together with that for *D10S1483*, indicates with very high probability that the double nucleotide substitutions present in the two families have independent mutational origins.

## DISCUSSION

Pfeiffer syndrome is a rare autosomal dominant condition combining both craniofacial and limb anomalies. There is significant phenotypic variation, ranging from mild cases with no synostosis to severe cases with a cloverleaf skull shape. Mutation hotspots in the FGFR2 protein account for the majority of cases. The IgIII domain is the most frequently involved site, but the IgII domain, as in this newly presented British family, has also been implicated. The p.Ala172Phe mutation, involving a double nucleotide substitution, is very rare as it has only been reported once before, in Pfeiffer's original index pedigree [Pfeiffer, 1964; Kan et al., 2002].

In these two families, the Pfeiffer syndrome phenotype associated with the p.Ala172Phe mutation is atypical in both its combination of clinical signs and in its severity. This mutation results in a “milder” cranial phenotype; the most striking feature of which is a normal cranial suture pattern, with absence of craniosynostosis. We compared the phenotypes of the British family seen in our clinic and four affected members of the German family reported in detail in Pfeiffer's [1964] index paper (Table 1). None of the British family had craniosynostosis. In the original German family, the proband, a boy born in 1961, did not have craniosynostosis; similarly, his father, paternal uncle, and paternal aunt had normal, open cranial sutures. Despite the absence of craniosynostosis, other craniofacial anomalies were consistently present. Brachycephaly was a relatively frequent sign. Of the British family, only the proband had brachycephaly and this was mild; the CI was 85%, slightly above the normal range of 76–83% [Haas, 1952]. However, brachycephaly was described in all affected members of the German family. Midface hypoplasia was a feature described in all except the proband in the British family, and all members of the German family. Hypertelorism was present in all cases, British and German, except in the German proband's paternal aunt. A high arched hard palate was also seen in all patients, British and German, without exception. Additional subtle facial features were also described in both families; in the British family—dysmorphic facies featured across the generations, in the German family—exorbitism, divergent strabismus and low-set ears were variably reported. Concerning the limb anomalies, these are a more severe feature of the p.Ala172Phe mutation than most other FGFR2-associated Pfeiffer syndrome mutations. In the upper limbs of the British family, broad radially deviated thumbs and a variable pattern of syndactyly were present in all generations. In the German family, all members were reported to have broad radially deviated thumbs, but syndactyly was only seen in the proband and his father and not seen in the paternal aunt and uncle. In both families, all those who had radiographs taken of their feet had absence of the toe middle phalanges. Comparison of the unrelated families demonstrates that although there is variable expressivity, overall, there is tight genotype–phenotype (mild skull/severe limb) correlation with the p.Ala172Phe mutation, both within and across families.

**TABLE I:** Comparison of the Phenotypic Characteristics of the British and German Families Showing Significant Similarities

	British family			German family*			
		
Phenotypic feature	Proband	Mother	Maternal grandfather	Proband	Father	Paternal uncle	Paternal aunt
Craniofacial							
**No craniosynostosis**	•	•	•	•	•	•	•
Brachycephaly	•			•	•	•	•
Midface hypoplasia		•	•	•	•	•	•
Dysmorphic facies	•	•	•	•			
Hypertelorism	•	•	•	•	•	•	
Flattened nasal bridge			•	•			
Strabismus				•	•		•
**High arched palate**	•	•	•	•	•	•	•
Limb—upper							
**Broad radially deviated thumb**	•	•	•	•	•	•	•
3/4 or 2/3/4 syndactyly	•	•	•	•	•		
Little finger clinodactyly	•						
Limb—lower							
**Broad medially deviated great toe**	•	•	•	•	•	•	•
Other broad toes		•	•	•			
2/3 or 2/3/4 or 2/3/4/5 syndactyly	•	•	•	•	•		
Absent middle phalanges	•	INA	INA	•	•	•	INA

•, identified in subject; *, individuals reported in detail in the original article [Pfeiffer, 1964]; INA, information not available; bold type, feature present in all cases in both British and German families.

Our analysis using a nearby informative microsatellite and SNP, which flank the site of the mutation, provide strong evidence for independent mutational origins in the German and British families. The alternative hypothesis of a single mutational origin would require either secondary mutation of both the microsatellite and SNP, or mutation of the microsatellite and recombination between the mutation and the SNP. Analysis of phased data from HapMap showed that the haplotypes to which we assigned each mutation (German G-A-C-T-C, British G-A-C-T-T) are both present at measurable frequency in the CEPH-Utah population (24/234 and 6/234 chromosomes, respectively). By comparison the genome-averaged probability of recombination within a physical distance of 15.7 kb (assuming 1 Mb ≍ 1 cM) would be ∼1.6 × 10^−4^ per meiosis, giving a lower relative probability of recombination even if dozens of meioses separated an ancestral mutational event between the two families, compared to independent mutations on two different haplotypes.

Two features of the conclusion that the two families have independent mutational origins appear remarkable—first, that a double nucleotide mutation should occur within the exon encoding the IgII domain when no single nucleotide mutation affecting this domain has been recorded; and second, that this identical double nucleotide mutation should occur independently in two different families. However, both observations can be rationalized based on the known biology and pathophysiology of FGFR2 action. Upon binding of the FGF ligand, two adjacent FGF-FGFR complexes dimerize with activation of the tyrosine kinases. The dimer, shown to involve a symmetric and two-ended configuration [Ibrahimi et al., 2005], is stabilized by both ligand–receptor interactions and direct receptor–receptor contacts. In the p.Ala172Phe mutation, substituting the small alanine side chain to bulky phenylalanine introduces an additional hydrophobic contact between mutant receptor pairs, through stacking of the aromatic side chains of the phenylalanine residue, with uniquely enhanced stabilization and consequent gain-of-function. Owing to the characteristics of the genetic code, this outcome is only possible through the simultaneous mutation of two nucleotides in the p.Ala172 codon. This very specific structural mechanism predicts that other mutations in IgII, including the several different amino acid substitutions that could arise from single nucleotide mutations at the alanine 172 codon, will not have the same pathophysiological effect.

A seeming paradox raised by our conclusion that the same double nucleotide mutation has arisen independently on two separate occasions, is that a random double nucleotide substitutions arising by chance alone are expected to be present less than once in the entire human population [Kondrashov, 2002]. This paradox can be explained by invoking the process of selfish spermatogonial selection leading to the phenomenon of paternal age effect (PAE) mutation, for which *FGFR2* provides a paradigmatic example [Goriely and Wilkie, 2012]. Such mutations are predicted to become slowly enriched by clonal expansion over many years, because of a selective advantage conferred to the spermatogonial cell in which they arise; this mechanism can increase the level of mutations in sperm by several orders of magnitude above the background mutation rate. A consequence of this process is that recurrent instances of particular multiple nucleotide substitutions that confer specific gain-of-function characteristics to the encoded protein may be observed—examples of such independent multinucleotide mutations have been described in several PAE genes including *FGFR2* [Goriely and Wilkie, 2012].

In conclusion, Pfeiffer syndrome resulting from the p.Ala172Phe mutation is infrequent—only two families, 500 miles and 45 years apart. However, the independent origin of the two double nucleotide substitutions, and similar phenotypes associated with the resulting missense mutation, lend weight to the exquisite specificity of the functional consequences of this particular mutation.
